# Evaluation of surgical skill using machine learning with optimal wearable sensor locations

**DOI:** 10.1371/journal.pone.0267936

**Published:** 2022-06-03

**Authors:** Rahul Soangra, R. Sivakumar, E. R. Anirudh, Sai Viswanth Reddy Y., Emmanuel B. John

**Affiliations:** 1 Department of Physical Therapy, Crean College of Health and Behavioral Sciences, Chapman University, Irvine, California, United States of America; 2 Department of Electrical and Computer Science Engineering, Fowler School of Engineering, Chapman University, Orange, California, United States of America; 3 Department of Sensor and Biomedical Technology, School of Electronics Engineering, Vellore Institute of Technology, Vellore, India; Universiti Malaysia Pahang, MALAYSIA

## Abstract

Evaluation of surgical skills during minimally invasive surgeries is needed when recruiting new surgeons. Although surgeons’ differentiation by skill level is highly complex, performance in specific clinical tasks such as pegboard transfer and knot tying could be determined using wearable EMG and accelerometer sensors. A wireless wearable platform has made it feasible to collect movement and muscle activation signals for quick skill evaluation during surgical tasks. However, it is challenging since the placement of multiple wireless wearable sensors may interfere with their performance in the assessment. This study utilizes machine learning techniques to identify optimal muscles and features critical for accurate skill evaluation. This study enrolled a total of twenty-six surgeons of different skill levels: novice (n = 11), intermediaries (n = 12), and experts (n = 3). Twelve wireless wearable sensors consisting of surface EMGs and accelerometers were placed bilaterally on bicep brachii, tricep brachii, anterior deltoid, flexor carpi ulnaris (FCU), extensor carpi ulnaris (ECU), and thenar eminence (TE) muscles to assess muscle activations and movement variability profiles. We found features related to movement complexity such as approximate entropy, sample entropy, and multiscale entropy played a critical role in skill level identification. We found that skill level was classified with highest accuracy by i) ECU for Random Forest Classifier (RFC), ii) deltoid for Support Vector Machines (SVM) and iii) biceps for Naïve Bayes Classifier with classification accuracies 61%, 57% and 47%. We found RFC classifier performed best with highest classification accuracy when muscles are combined i) ECU and deltoid (58%), ii) ECU and biceps (53%), and iii) ECU, biceps and deltoid (52%). Our findings suggest that quick surgical skill evaluation is possible using wearables sensors, and features from ECU, deltoid, and biceps muscles contribute an important role in surgical skill evaluation.

## Introduction

Surgical skills and techniques are central components of a surgeon’s skill set and directly correlate with patient benefits [1]. It is critical to assess surgical skills among trainees in surgical specialties to identify their competence and confidence to practice independently [2]. Surgical skills are influenced by decreasing work hours and increasing sub-specializations focusing on minimally invasive surgeries (MIS). These changes impact residency training since there are concerns for current surgery residents’ adequate operative experience and technical training [3, 4]. To determine the level of surgical skills and clinical competency, a multitude of surgical skill assessment tools, consisting of experts’ evaluation of surgical skill videos, which is often costly and time-consuming [5, 6] are used.

Furthermore, evaluation by experts often lacks objective data and may sometimes be biased by the reviewer [7, 8]. Thus, there is a lack of objective high-fidelity tools to evaluate surgical skills in hospital environments. Previously researchers have estimated surgical skills using i)kinematic data and convolutional neural network [9], ii) kinematic data as putative markers and deep neural networks [10], iii) virtual reality spinal task and machine learning algorithms (support vector machines, k-nearest neighbors, least discriminant analysis, naïve bayes and decision tree) [11], iv) image processing and deep neural network during robotic surgery [12–14], v) kinematic data from da Vinci robot and global rating score and machine learning (kNN, logistic regression, SVM) [15]. Recently deep learning-based haptic guidance systems have been used for surgical skill development [16]. Moreover, it is unknown which features from wearable sensors such as EMG and accelerometers could contribute more to skill identification. It is also unknown which location on the upper extremity can be helpful for accurate skill classification with minimal hindrance. Instead of evaluating surgical time, one needs to assess objective trajectorial movement and muscle activity features for correct skill classification and surgical ergonomics. In surgical skill evaluations, some studies evaluated the muscular workload during surgical procedures using surface electromyography (sEMG) (17–20) for muscle activity. Other researchers have utilized wearable inertial sensors consisting of accelerometers to determine efficiency between experts and trainees performing the same surgical task [17]. To some extent, both movement accelerations and muscle activities have shown the potential to distinguish subtle details on successfully identifying the skill level of surgeons. EMG signals are a reliable measure for physiological stress detection in the laboratory [18], and earlier studies have reported the upper trapezius muscle as an essential stress indicator [19, 20].

Nonlinear movement variability features can quantify neuromuscular connections (feedback) and subtle movement changes [21–23]. Researchers have reported that entropy measures such as approximate entropy, sample entropy, and multiscale entropy can estimate specific feedback mechanisms and spontaneous properties of interconnected neurons and are characterized as regularity [24–26]. Both linear and nonlinear features can be extracted from EMG and accelerometer sensors, respectively. Feature extraction is crucial for accumulating information relevant to surgical skill evaluation. The selection of feature vectors from EMG and accelerometer signals during surgical tasks needs careful consideration since many muscles and features may contain redundant information [27, 28]. This study extracted muscle work-related EMG features and linear and nonlinear movement variability features from accelerometer signals. Appropriate features will result in higher classification accuracy with maximum skill separability and robustness [27, 28]. With recent developments in machine learning, we will explore critical objective biomarkers for identifying surgical skillsets with high accuracy; however, there are multiple sites where wearable sensors such as EMG and inertial sensors can be affixed. This study will consider the performance analysis for feature extraction and selection algorithms from two fundamental perspectives: 1) Which features varied significantly among three surgical tasks and best differentiate surgical skillset? 2) Which sensor position offers the skill classification. This study will attempt to establish the groundwork for identifying i) important sites of sensor placement and ii) important linear and nonlinear features which can distinguish skill levels amongst residents, medical students, and expert surgeons. Our results will help develop wearable devices integrated with machine learning technologies to evaluate surgeons’ performance during minimally invasive surgical tasks.

## Materials and methods

**Fig 1:**
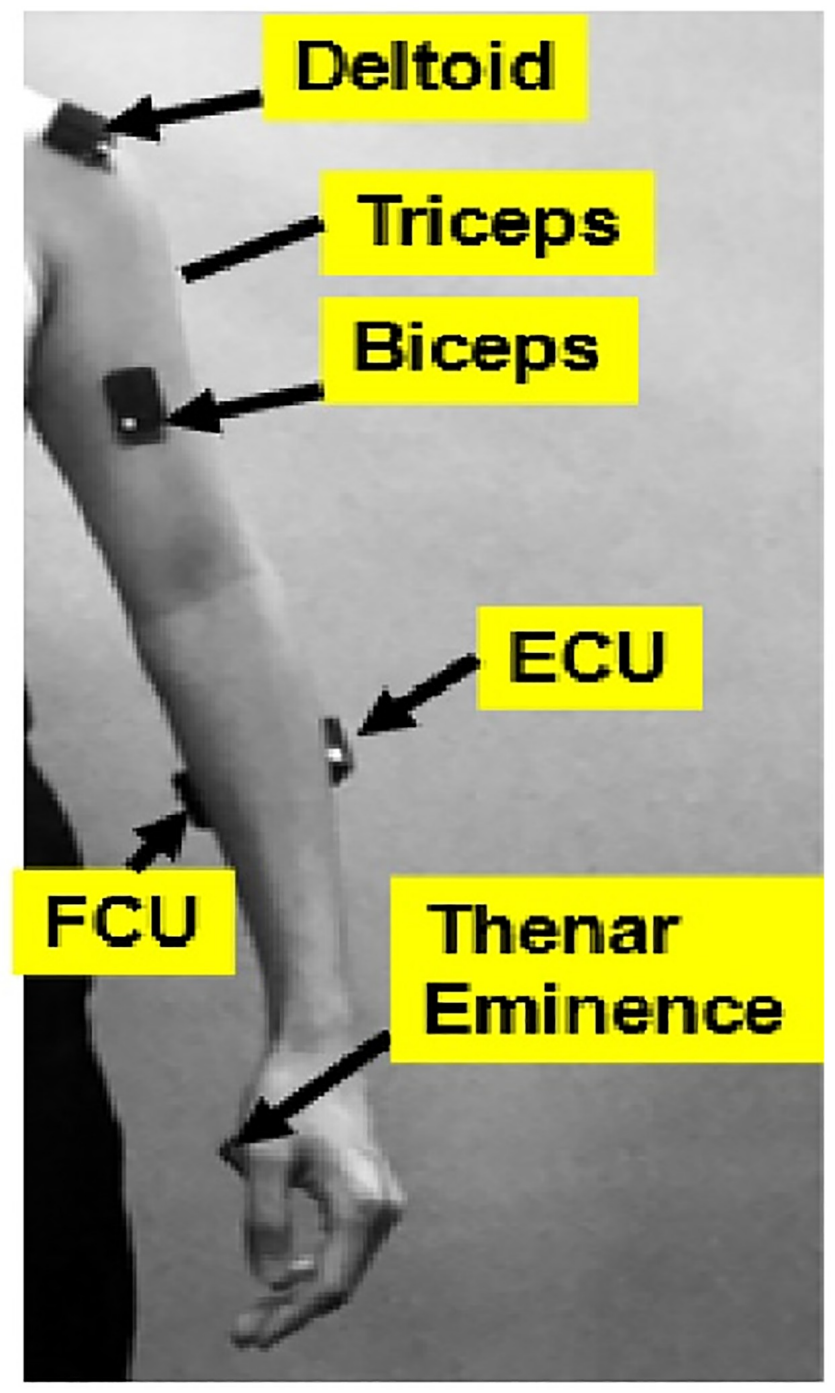
Muscle sites for Delsys Trigno EMG sensor attachment. The muscle sites included deltoid, triceps, biceps, Extensor Carpi Ulnaris (ECU), Flexor Carpi Ulnaris (FCU), Thenar Eminence (TE) for the left and right side.

Twenty-six participants categorized with experience from the Department of Urology at the University of California, Irvine, (UCI) participated in this study. All participants signed the written consent form approved by UCI (HS#: 2018–4407). The three groups were categorized based on their surgical experience; i) novice (undergraduate or medical students without prior surgical experience) (N = 10), ii) intermediate (urology residents postgraduate year 1–5) (N = 11), and expert surgeons (urology physicians with more than five years experience) (N = 5). Participants performed basic surgical tasks; i) pegboard transfer, ii) knot tying, and iii) robotic suturing. At least three trials were conducted for each surgical task. The participants were asked to perform these tasks at their normal pace. To identify optimal locations for surgical workload assessment, electromyography (EMG) sensors with in-built accelerometers were used to estimate muscular activation level and timing. A total of twelve EMG surface electrodes (DELSYS^®^ Trigno^™^ Wireless, Boston, MA) were attached bilaterally on bicep brachii, tricep brachii, anterior deltoid, flexor carpi ulnaris (FCU), extensor carpi ulnaris (ECU), and thenar eminence (TE) (Fig 1). Resting data was collected during sitting and standing postures. These muscle groups were selected since previous studies on surgical ergonomics have highlighted their importance and activations [29]. Deltoid was selected since the shoulder is the most common site for musculoskeletal symptoms reported by laproscopic surgeons [30]. Muscle activities were normalized using Maximum Voluntary Contraction (MVC) of each muscle group.

### Surgical skills tasks

**Fig 2:**
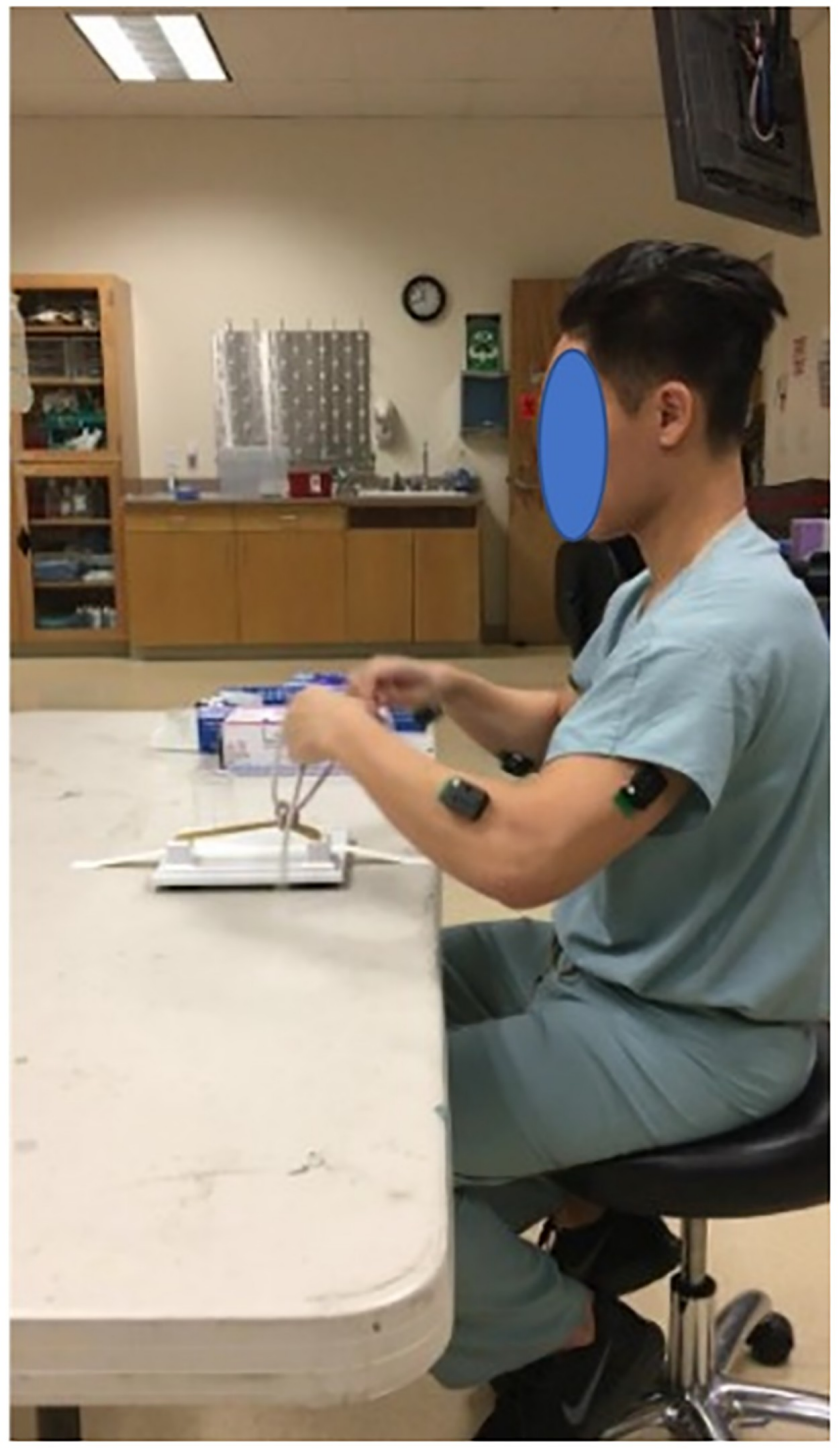
Knot tying task performed by participants.

**Fig 3:**
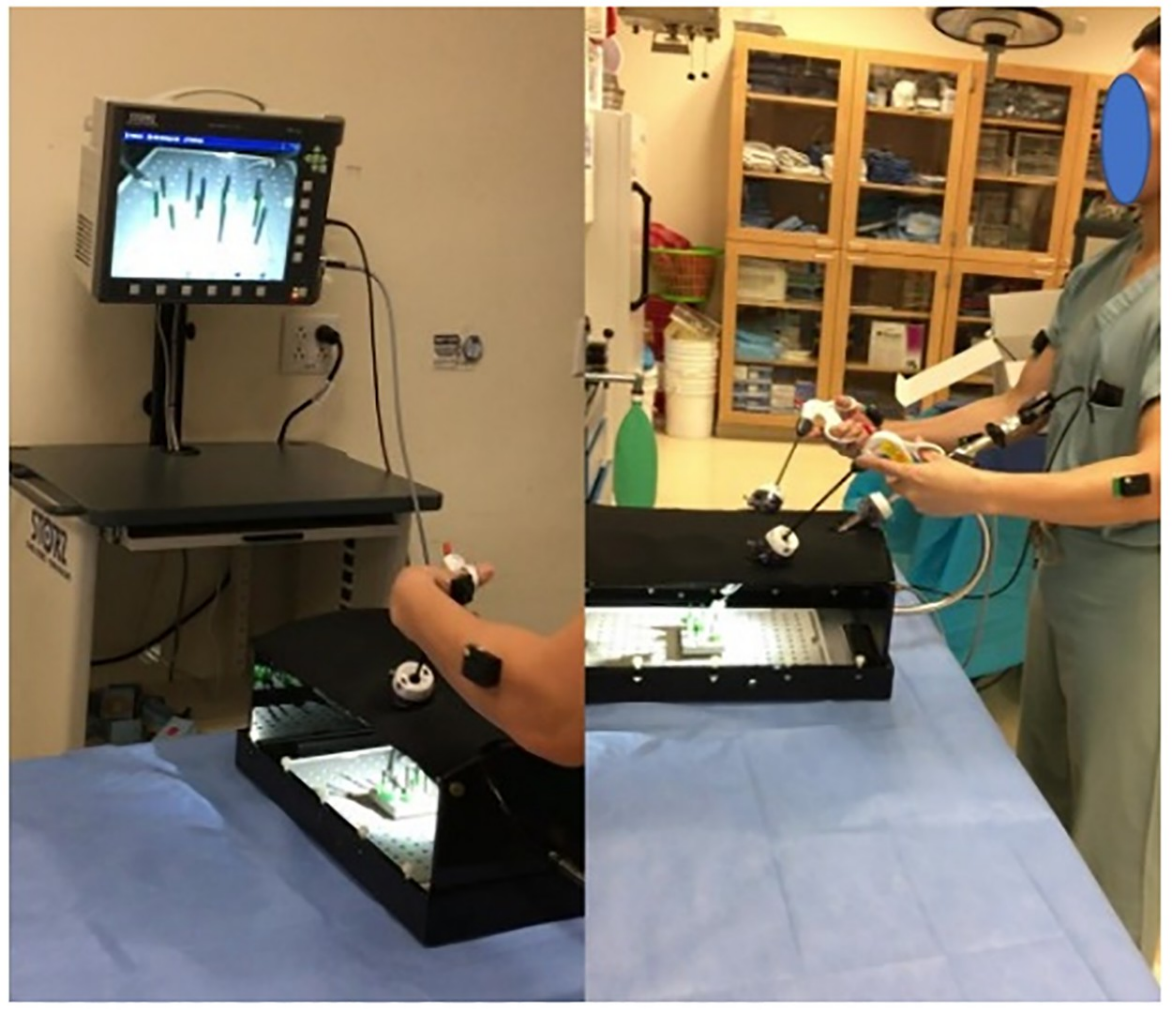
Pegboard transfer task performed by participants.

**Fig 4:**
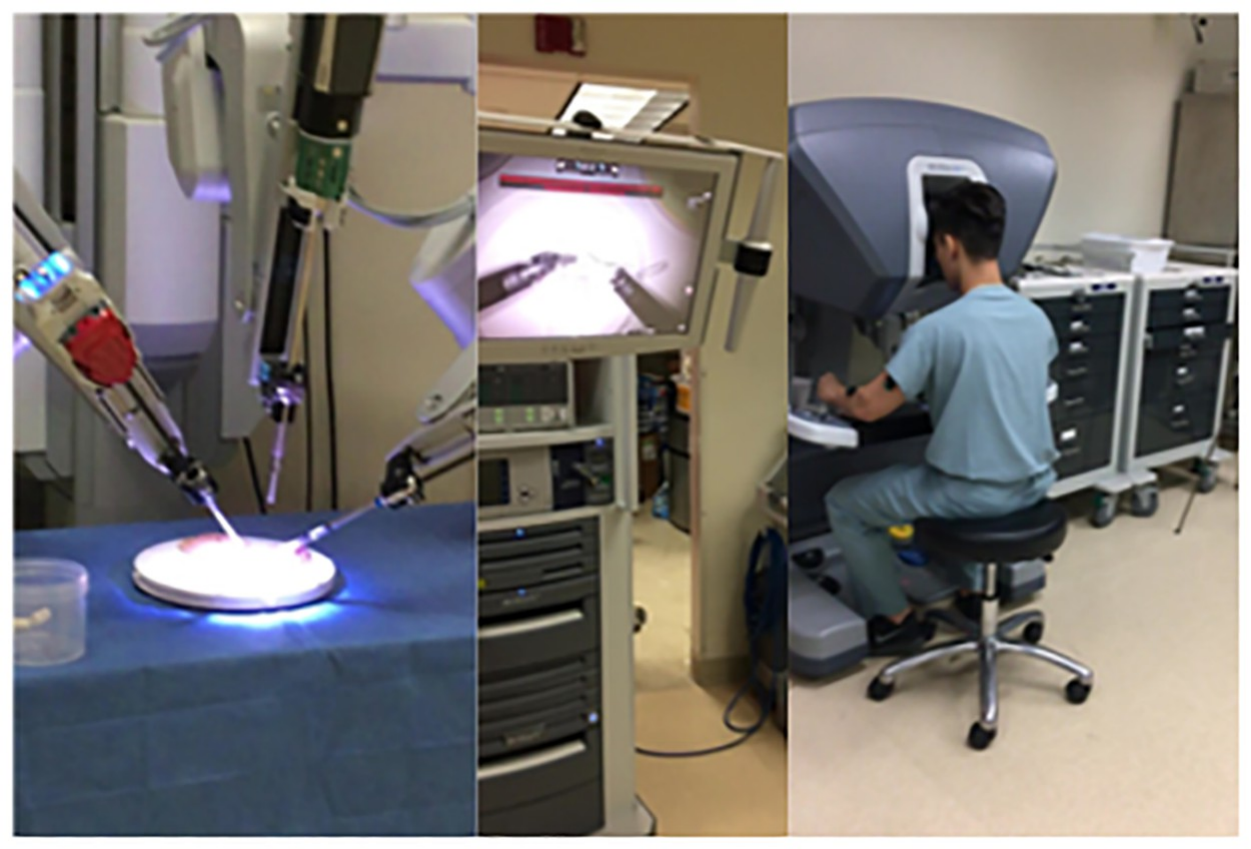
(a) position of six muscles sites where surface EMG sensors were affixed (b)Pegboard transfer task (c) Robotic suturing task using Da Vinci (d) Knot tying task (e) Ureteroscopy task.

All participants performed four surgical tasks. i) *Open knot tying*: Two-handed surgeon’s knot with other three-square knot throws was tied over two rubber bands mounted on a knot tying practice board (Ethicon Inc., Somerville, NJ) (Fig 2). ii) *Laparoscopic peg transfer*: The task aims to transfer the objects (rubber pegs) from one side of the board to the other and back using two laparoscopic graspers. Six pegs are placed on the left side of the board; each peg is picked with the left grasper, transferred midair to the right grasper, and then placed over a post on the right side of the board. Once all pegs have been transferred, the process is reversed (Fig 3). Laparoscopic peg transfer was completed on an SZABO-BERCI-SACKIER Laparoscopic Trainer (Karl Storz, Tuttlingen, Germany). iii) *Robotic suturing*: A needle is passed through a rubber Penrose drain using da Vinci^®^ Si surgical system robotic needle drivers (Intuitive, Sunnyvale, CA). The instrument knot is then reinforced with three additional square knots (Fig 4). EMG data were recorded at 2000 Hz, and accelerometer data were sampled at 100Hz. For EMG processing, raw signals were detrended to remove bias. EMG signals were then band-pass filtered from 20–500 Hz followed by a notch filter of 60 Hz to attenuate 60Hz electrical noise. Filtered signals were rectified using Root Mean Square (RMS) utilizing a window size of 6 data points and filtered using a fourth-order low-pass filter with a cutoff frequency of 5 Hz.

### EMG preprocessing and feature extraction

**i) Muscular workload-related features**: Cumulative muscular workload represents the total amount of work for each muscle group, normalized for MVC, as a function of time. The average muscular workload was computed as the total cumulative work done per second. **ii) Time-domain feature**: The time-domain features are quick and easy to compute [31–33]. The EMG signals were rectified using windowed RMS and then low-pass filtered. The muscle activation time was computed using a threshold from baseline data and five standard deviations, as shown in Table 1 below. Total time of muscular activity was computed when muscle onset and muscle cessation points were determined, **iii) linear variability features**: variability features such as signal range and RMS were evaluated for quantifying muscle firing variability during a task. **iv) frequency-domain feature**: dominant muscle firing frequency was evaluated for each muscle.

**Table 1:** Temporal and frequency domain features extracted from EMG signals.

Features	Feature Evaluation Method
**Cumulative Muscular Workflow** (CMW) was computed through the integration of normalized EMG data for time throughout the task	$Cumulative\;muscular\;workload\;\left(CMW\right)=\overset{t}{\underset{0}{\int }}\frac{Rectified\;EMG}{MVC}$
**Average Muscular Work done per second**	$Average\;Work\;per\;Second\;\left(AWS\right)=\frac{CMW}{Performance\;Time}$
**Total Time**: Total time was evaluated utilizing a threshold; start time was recorded when EMG activity was more than the threshold.	*Threshold* = *Mean* + 5 *X SD*
**RMS**: Root means square measures linear variability through quantifying fluctuations in a signal, where n is the length of the EMG signal.	$EMG_{RMS}=\sqrt{\frac{1}{n}\sum_{i=1}^{n}EMG_{i}^{2}}$
**Signal Range**: Signal range was computed as the difference between the maximum and minimum EMG activity	Range = EMG_max_ − EMG_min_
**Signal Frequency**: The dominant frequency carries more energy to other frequencies on the EMG spectrum.	EMG signal is detrended zero-padded to the nearest higher power of 2 Fast Fourier transform (FFT) was computed Modulus of signal FFT (complex number) is squared to get raw power spectral density (PSD) The frequency with the highest amplitude in the PSD is the dominant frequency of the EMG signal.

### Accelerometer feature extraction

Both linear and nonlinear features were extracted from accelerometer data, as shown in Table 2 below.

**Table 2:** Linear and nonlinear features extracted from accelerometer data.

Features	Feature Evaluation Method
**Resultant Acceleration Timeseries**: A time series was extracted from each sensor using the resultant of 3-dimensional acceleration as given by equation 1.	$R_{Acc}=\sqrt{Acc_{X}^{2}+Acc_{Y}^{2}+Acc_{Z}^{2}}\;\;(1)$ Acc_x_, Acc_y_, and Acc_Z_ are acceleration in X, Y, and Z-directions.
**Correlation Dimension**: The correlation dimension was introduced by Grassberger and Procaccia [34]. The correlation dimension was computed from resultant acceleration and is a fractal dimension unrevealing system’s complexity [35, 36] and quantifies inherent dynamical behaviors with a single number.	For an m-dimensional phase space, the correlation function C(r) is given by equation 2 below. $C\left(r\right)=\underset{N\to \infty }{\text{lim}}\frac{2}{N\left(N-1\right)}\sum_{\begin{array}{c} i,j\\ \left(1\leq i<j\leq N\right) \end{array}}H\left(r-\left|Y_{i}-Y_{j}\right|\right)\;\;(2)$ Where H is the Heaviside step function, with H(u) = 1 for u>0, and H(u) = 0 for u≤0, where u = r-|Y_i_-Y_j_|, r is the radius of a sphere centered on Y_i_ or Y_j_, and N is the number of data points.
**Multiscale Entropy (MSE)**: Entropy techniques effectively quantify the probability that neighboring points in the resultant acceleration time series will be within a predetermined range. Entropy is a measure of complexity in physiological systems denoting a highly adaptable network of neuromuscular connections attained by regular practice [37, 38]; increased MSE is indicative of a greater degree of complex movement dynamics [38, 39].	Utilizing the coarse-graining process to time series, new time series is constructed by averaging the data points within non-overlapping windows of increasing length, τ. Each time series element y_j_^(τ)^, is given by equation 3. $y_{j}^{\left(\tau \right)}=\frac{1}{\tau }\sum_{i=\left(j-1\right)\tau +1}^{j\tau }x_{i}\;\;(3)$ Where τ represents the scale factor and 1≤ j ≤ N/τ. The length of each coarse-grained time series is N/τ.
**Rosenstein’s Lyapunov Exponent**: Lyapunov exponents can detect the presence of chaos in a dynamical system by quantifying divergence in trajectories [40]. Resultant acceleration time series from sensors were utilized to assess nonlinear variability and chaotic properties.	i) A delayed reconstruction Y_1:N_ with embedding dimension ‘m’ and lag ‘τ’. ii) For a point ‘i’, the algorithm finds the nearest point i* that satisfies min||Y_i_-Y_i*_|| such that |i-i*|> ‘Minimum Separation,’ where ‘Minimum Separation,’ the mean period, is the reciprocal of the mean frequency. iii) The Lyapunov Exponent for the entire expansion range is calculated as in equation 4, $\lambda \left(i\right)=\frac{1}{\left(K_{max}-K_{min}+1\right)dt}\sum_{K=K_{min}}^{K_{max}}\frac{1}{K}\;\text{ln}\;\frac{\left\|Y_{i+K}-Y_{i*+K}\right\|}{\left\|Y_{i}-Y_{i*}\right\|}\;\;(4)$ Kmin and Kmax represent Expansion Range, ‘dt’ is the sampling time and divergence at ith point is given by equation 5. $ldiv=\text{ln}\;\frac{\left\|Y_{i+K}-Y_{i*+K}\right\|}{\left\|Y_{i}-Y_{i*}\right\|}\;\;(5)$ iv) A single value for the Lyapunov exponent is then computed from the earlier step using polynomial fit as given in equation 6 $LyE=polyfit\left(\left[K_{min}\;K_{max}\right],\;\lambda \left(i\right)\right)\;\;(6)$

### Feature Selection (FS)

Feature selection algorithms are necessary since they can remove redundant or unnecessary information from features that do not improve classification accuracy and help lower computational costs [41]. The best subset of features is associated with sensors placed at different muscles. Feature selection was utilized to reduce the number of input features (dimension reduction) or remove the redundant features. This dimension reduction allows lower computing speed and even reduces space complexity. We explored filter and wrapper feature selection methods such as Correlation-based Feature Selection (CFS) and Recursive Feature Elimination (RFE). CFS shows how well two features are correlated with a value ranging from -1 to 1, whereas RFE provides a ranking of features by giving weights to a particular part.

Filter feature selection methods are usually the first choice as it is not computationally expensive compared with wrapper and embedded feature methods. Filter methods are advantageous since they prevent the model from overfitting solely depending on the dataset’s statistical distribution.

### Correlation-based Filter Feature Selection (CFS)

CFS works well on supervised than unsupervised problems. It groups a subset of highly correlated features with the target attribute(i.e., novice, intermediate, expert). CFS measures the relationship between two variables with statistical data but is limited to identifying only the linear relationship between the features. It works on the principle that we could drop the features with lower correlation coefficients if the predictor variables are correlated. We evaluated pair-wise correlation among feature sets. The value of the correlation coefficient varies between +1 and -1. A value of ± 1 indicates a perfect degree of association between the two variables. As the correlation coefficient value goes towards 0, the relationship between the variables is weaker. Pearson correlation coefficients provide a relationship among features and use information about the mean and standard deviation from the data compared to nonparametric correlation, which only uses the ordinal information and scores of pairs. It is limited since it can only assess the linear relationship between the features. However, the correlation between the categorical attributes and the target attributes cannot be determined.

### Recursive Feature Extraction (RFE)

RFE is an external estimator and assigns weights to features. The principle is to select features by recursively considering a smaller set of features. The model is trained on the initial set of features, and with each iteration, we can calculate the feature coefficient. Then the least essential features will be removed, and then again, the model will be trained. Since it takes a lot of iterations to complete the whole ranking process, the computational time is quite expensive. Feature selection methods are performed on the entire muscle dataset, including different independent features to evaluate the surgeon’s skill in that particular task. This dataset accumulates data generated by six muscle sensors attached to the surgeon. Feature selection methods have been implemented in the Jupyter notebook with the help of pandas python library for ML. Reducing the features from 26 to 10 has increased the accuracy by five and reduced computational time.

### Model development

After feature selection, preprocessing techniques is used to transform the initial dataset to standardized values. Methods such as imputer and standardization are implemented to enhance the dataset’s quality. We trained three supervised ML models to assess the dataset: Random Forest Classifier (RFC) with 100 estimators, Naive Bayes classifier (NB) with kernel set to Radial Basis Function (RBF), Support Vector Machine (SVM) with all the default parameters. The objective of these models is to classify a surgeon’s skill based on the feature instances. For all the three supervised machine learning models, both train and test sizes were kept at the same ratio (70/30). We used 70% of the whole dataset for training and 30% for testing. According to the six muscle types, the entire dataset is divided into six sub-datasets. These six sub-datasets are then trained on three ML models to investigate which muscle is vital to identify the surgical skill. A few performance parameters were considered to evaluate the model to determine which model has done better on a particular sub-dataset. These parameters include precision, recall, f1 score (harmonic mean of precision and recall), true positive rate (TPR), false-positive rate(FPR). A **true positive (TP)** is an outcome where the model *correctly* predicts the *positive* class. Similarly, a **true negative (TN)** is an outcome where the model *predicts the negative class correctly*. A **false positive (FP)** is an outcome where the model *incorrectly* predicts the *positive* class. A **false negative(FN)** is an outcome where the model *incorrectly* predicts the *negative* class. The accuracy, recall, specificity, precision, and F1-score were evaluated using Eqs 7–11 below.


$$Accuracy=\frac{TP+TN}{TP+FP+TN+FN}$$ (7)

$$Recall=\frac{TP}{TP+FN}$$ (8)

$$Specificity=\frac{TN}{TN+FP}$$ (9)

$$Precision=\frac{TP}{TP+FN}$$ (10)

$$F1\;Score=\frac{2XRecall\;X\;Precision}{Recall+Precision}$$ (11)

### Supervised classifiers

Supervised learning algorithms are helpful in model correlation and the dependencies among their input features to predict the output values for new data based on the relationships learned from the training data sets. This study used supervised classification algorithms to classify three distinct skill groups—Novice, Intermediate, Expert.

### Feature selection process

**Fig 5:**
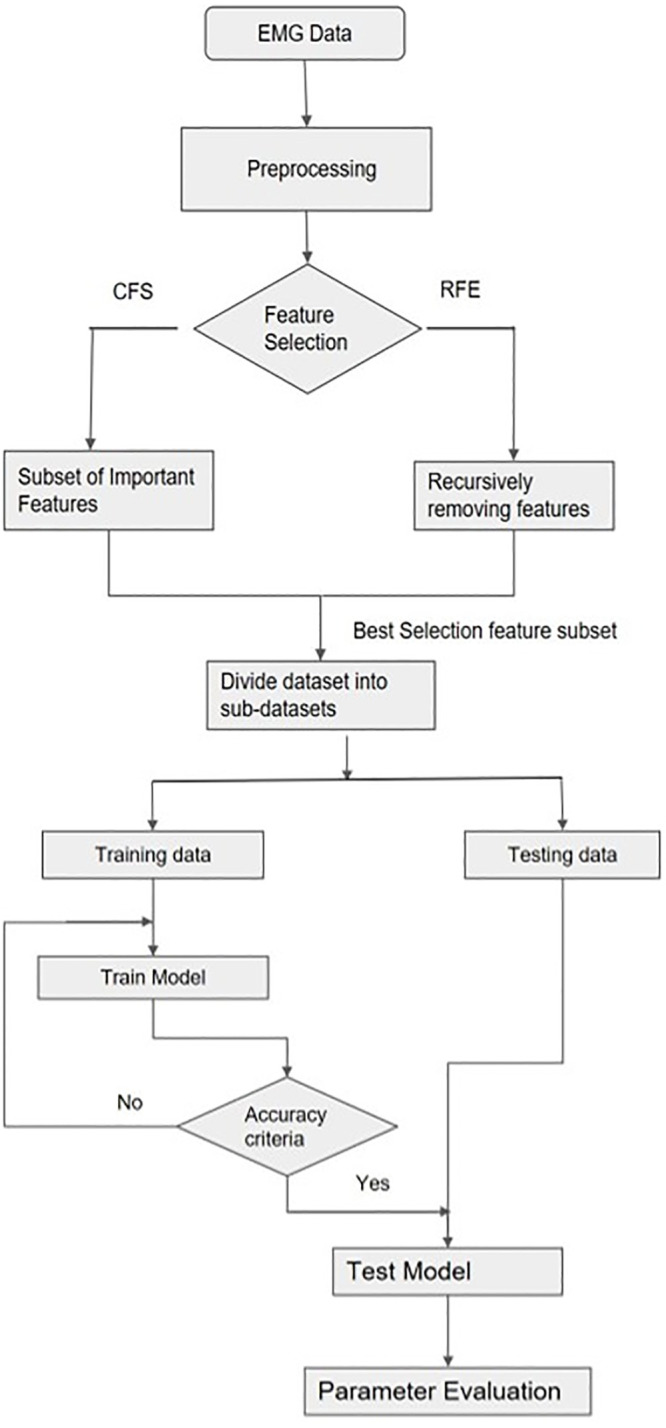
Flow chart of feature selection-based model development and identification of parameters of muscle importance for skill classification.

Preprocessing was conducted to remove impurities for higher model classification accuracy. Feature selection methods like CFS and RFE were used to identify features that could improve model accuracy significantly. The feature subset was divided into six subsets as per the muscle type. Each data subset was divided into training and testing sets (70/30), training data was used to train the 3 model types (SVM, RFC, and Naïve Bayes). The model parameters such as precision, recall, and accuracy were evaluated (Fig 5).

This study investigated muscles that could potentially classify surgical skills with high accuracy. The muscles that achieved better statistical performance than others were chosen, and others were dropped in evaluating the surgical skill. The priority of muscles from all models was assigned equal importance and weightage, and finally, the muscles that showed the highest significance for all three ML models were selected.

### Random Forest Classifiers (RFC)

Random Forest is a machine learning algorithm based on decision trees. Initially, all root nodes have different supervised classifiers, and each training data is predicted. The class selected as output for most by the classifiers is regarded as the final output by the RFC. RFC consists of multiple individual decision trees that operate as an ensemble. Each unique tree in the random forest divides a class of predictions, and the class with the most votes becomes the model’s prediction.

In this study, RFC was trained using input linear and nonlinear EMG and accelerometer features to predict which target class is more suitable for the new instance. The hyperparameters for the RFC are set to default parameters levels (Table provided in S1 Table). The feature data set was divided into 6 sub-datasets based on muscle attributes. In this dataset, we have six different muscles groups. The performance test scores attained by this model for surgical skill classification across these datasets were evaluated.

### Naïve Bayes

Naive Bayes is a rapid classification algorithm best suited for a massive chunk of data. It operates on the Bayes theorem of probability for the prediction of a new instance. The classification stage comprises two general phases: the learning and evaluation phases. The classifier trains its model on a given dataset in the learning phase, whereas the evaluation phase tests the classifier performance. This classifier is trained on our dataset and predominately predicts the probability of a class (surgical skill level) in the target attribute. The class with the highest chance will be considered for the particular instance. This model has been evaluated based on a few performance metrics such as precision, recall, and f1score.

### Support Vector Machines

Support Vector Machines(SVM) are efficient with smaller datasets compared to other supervised learning classifiers. SVM is beneficial when nonlinear features are involved; in such cases, feature correlation with model output cannot be mapped. SVM distinguishes different classes by constructing a hyperplane in a multidimensional plane. It maximizes the margin between the classes so that there is probably less chance of a new instance overlapping two classes. SVM is considered in our approach because of the dataset being multi-dimensional (more features). Confusion metrics have been used to evaluate our model’s performance. The results suggest that the model accurately recalls the true positives (correctly predicting the positive class). For further analysis, we have plotted the ROC curves to decide the suitability of this classifier with our dataset. It was inferred from the ROC curve that SVM classifiers outperformed other classifier models.

### Statistical analysis

An initial MANOVA examined 1) Total Time, 2) RMS, 3) Range, 4) Frequency, 5) CMW, 6) AWS as dependent variables, and 1) Skill Level (Expert, Intermediate, and Novice) and 2) Surgical Test (Knot Tying, Pegboard, Robotic suturing) as independent variables. An overall 3 X 4 (Skill Level X Surgical Task) multivariate analysis of variance (MANOVA) with repeated measures design was applied to all data to investigate skill level using EMG and accelerometry signals. The main and interaction effects were analyzed using JMP^®^ Pro (SAS Institute Inc., Cary, NC, 2020), with the significance level of p = 0.05.

## Results

Six linear EMG features based on temporal and frequency domains (CMW, AWS, total time, RMS, Signal Range, and dominant frequency) (Table 1) and eleven nonlinear variability features were extracted from accelerometer data (Table 2). Feature selection attributed to important features from the dataset, which enhanced the predictive model’s performance.

We found significant interaction effects among skill level and surgical test for total time(F(4,24) = 218, p<0.01), RMS(F(4,24) = 2.97, p<0.05), Range (F(4,24) = 5.01, p<0.01), frequency (F(4,24) = 337, p<0.01), CMW(F(4,24) = 14.8, p<0.01), AWS (F(4,24) = 2.49, p<0.05).

**Fig 6:**
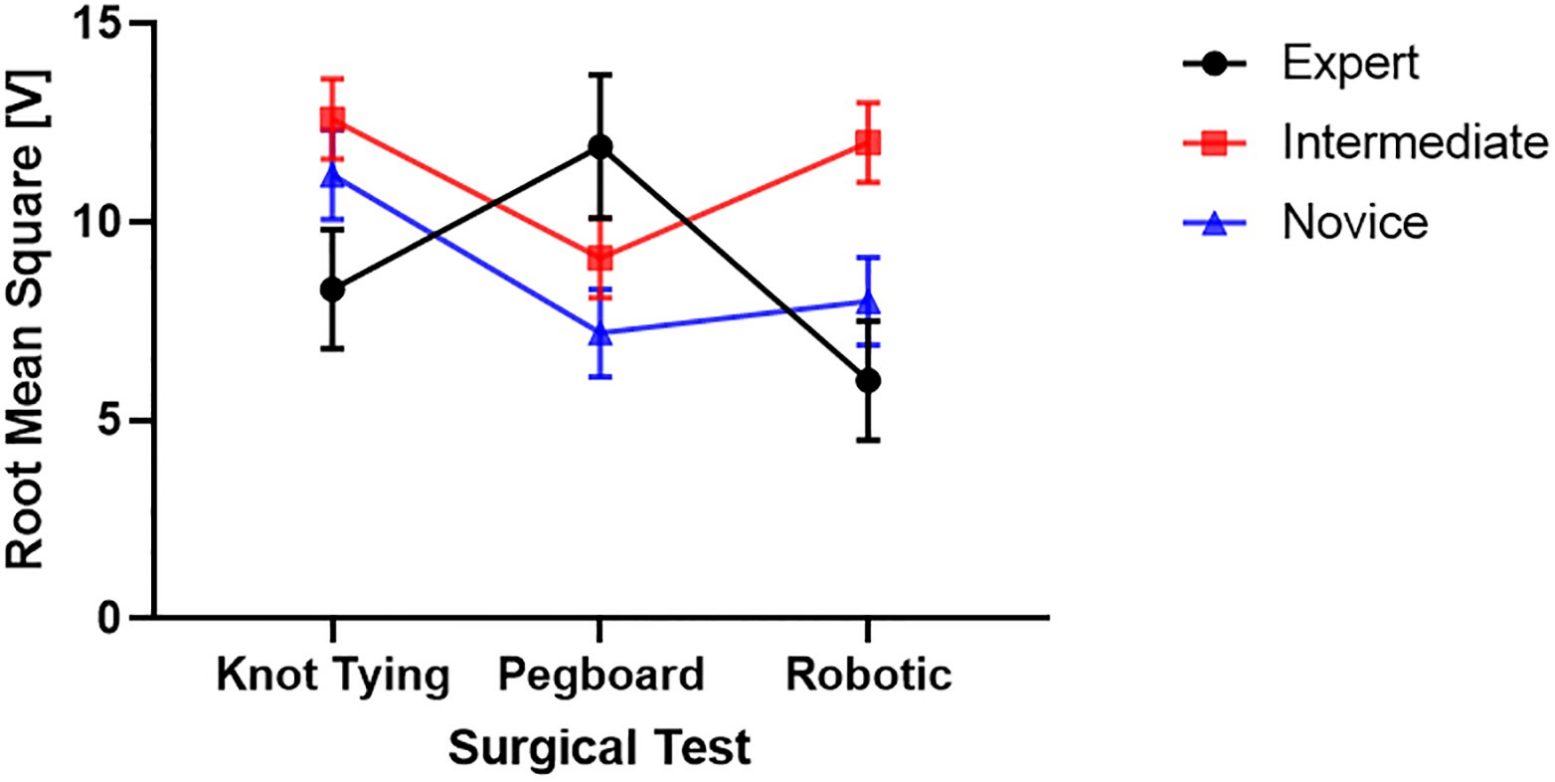
Linear variability as measured by RMS of EMG signals between groups of 3 skill levels.

Fig 6 shows experts showed fewer fluctuations in muscle firing (RMS) in knot tying and robotic suturing tasks. In contrast, there was somewhat higher variability during pegboard tasks than intermediate skilled and novice surgeons.

**Fig 7:**
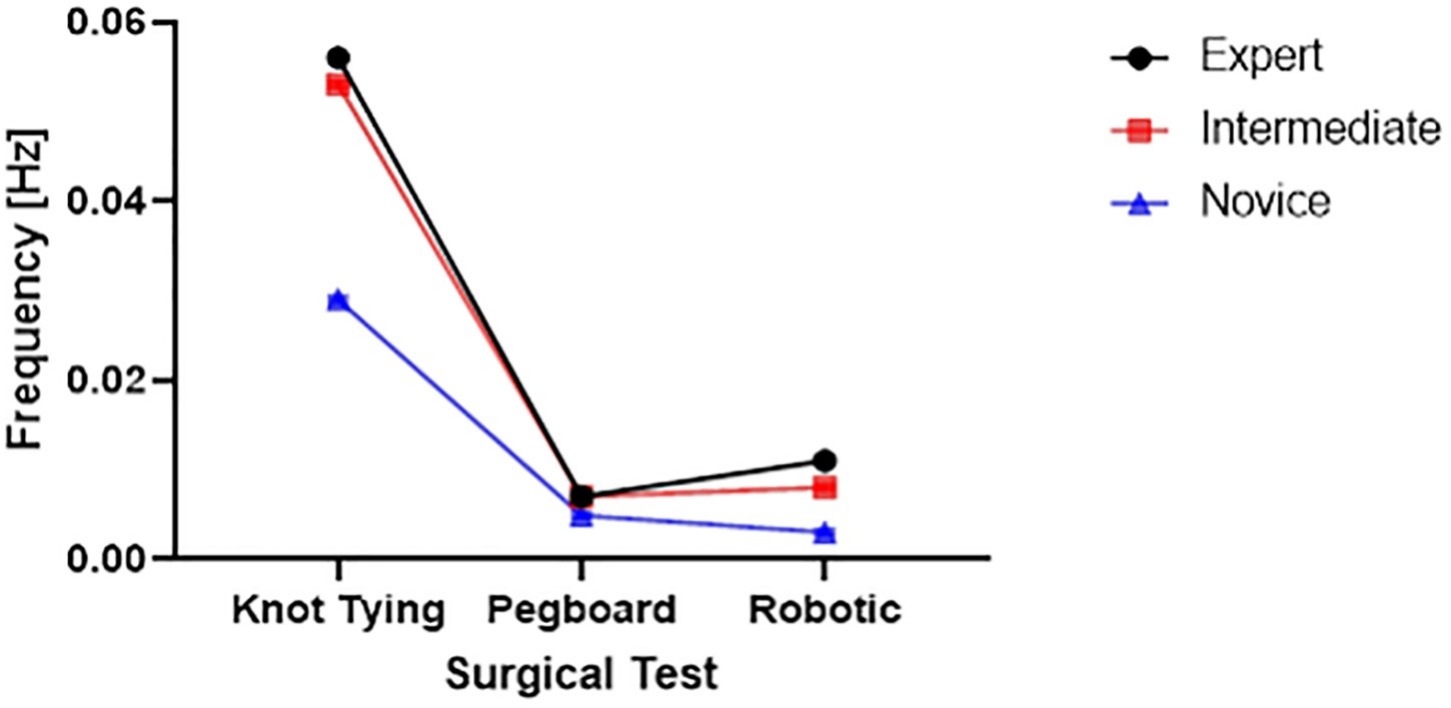
The dominant frequency of EMG signals during the performance of 3 surgical tasks (knot tying, pegboard transfer, and robotic suturing).

Frequency profiles of experts, novice, and intermediate skilled surgeons (Fig 7) show EMG signal dominant frequencies during the performance of three surgical tasks.

**Fig 8:**
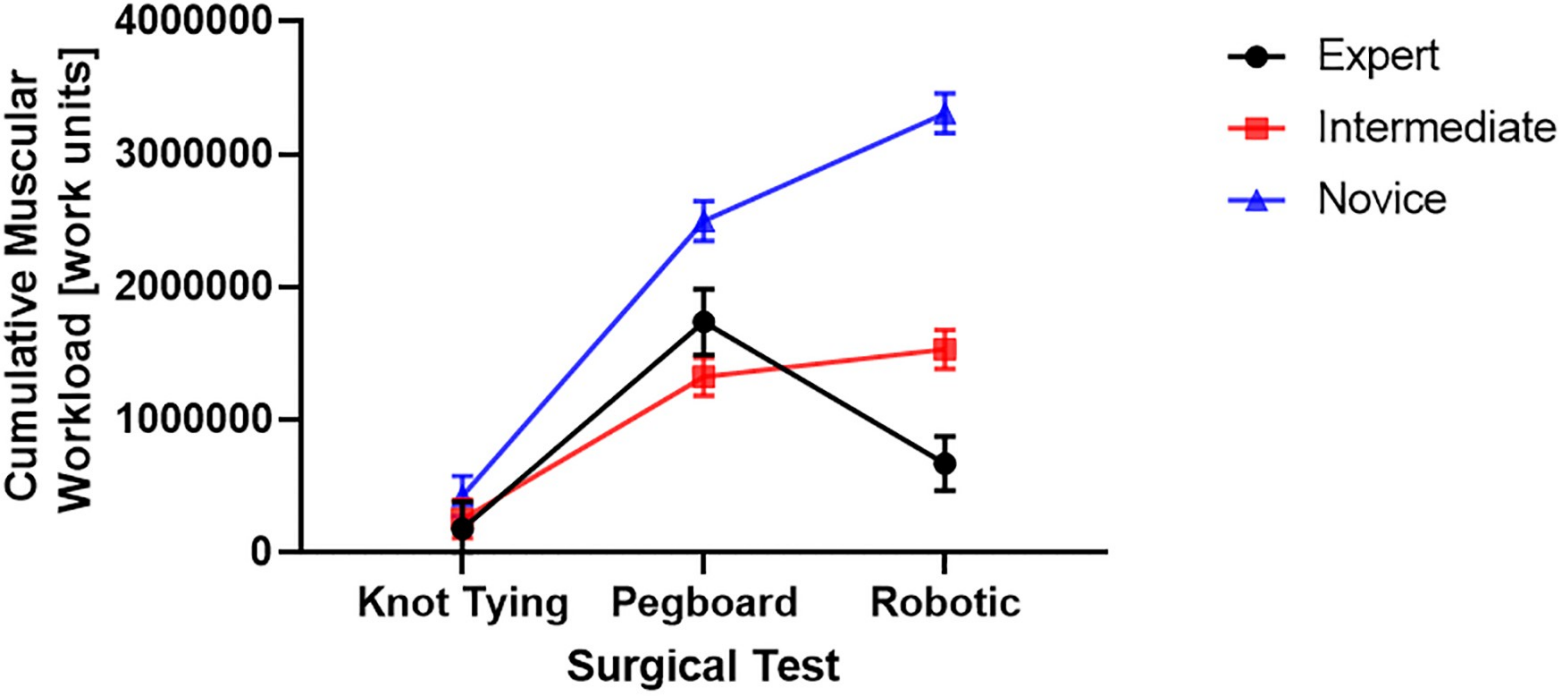
The cumulative muscular workload for completing surgical tasks (knot tying, pegboard transfer, and robotic suturing) by three groups of different skill levels (expert, intermediate, and novice).

We found that the cumulative muscular workload was higher for the novice group (Fig 8). Post hoc comparisons using the Tukey HSD test indicated that the CMW was significantly higher for the novice group than the intermediate group for pegboard (M = 2494810, SD = 149982 versus M = 1320467, SD = 143597) and robotic suturing (M = 3304601, SD = 149980 versus M = 1528405, SD = 145634) Fig 7. We also found that the expert group’s CMW (M = 669617, SD = 203076) was significantly lower than intermediate (M = 1528405, SD = 145634) and novice (M = 3304601, SD = 149980) groups.

**Fig 9:**
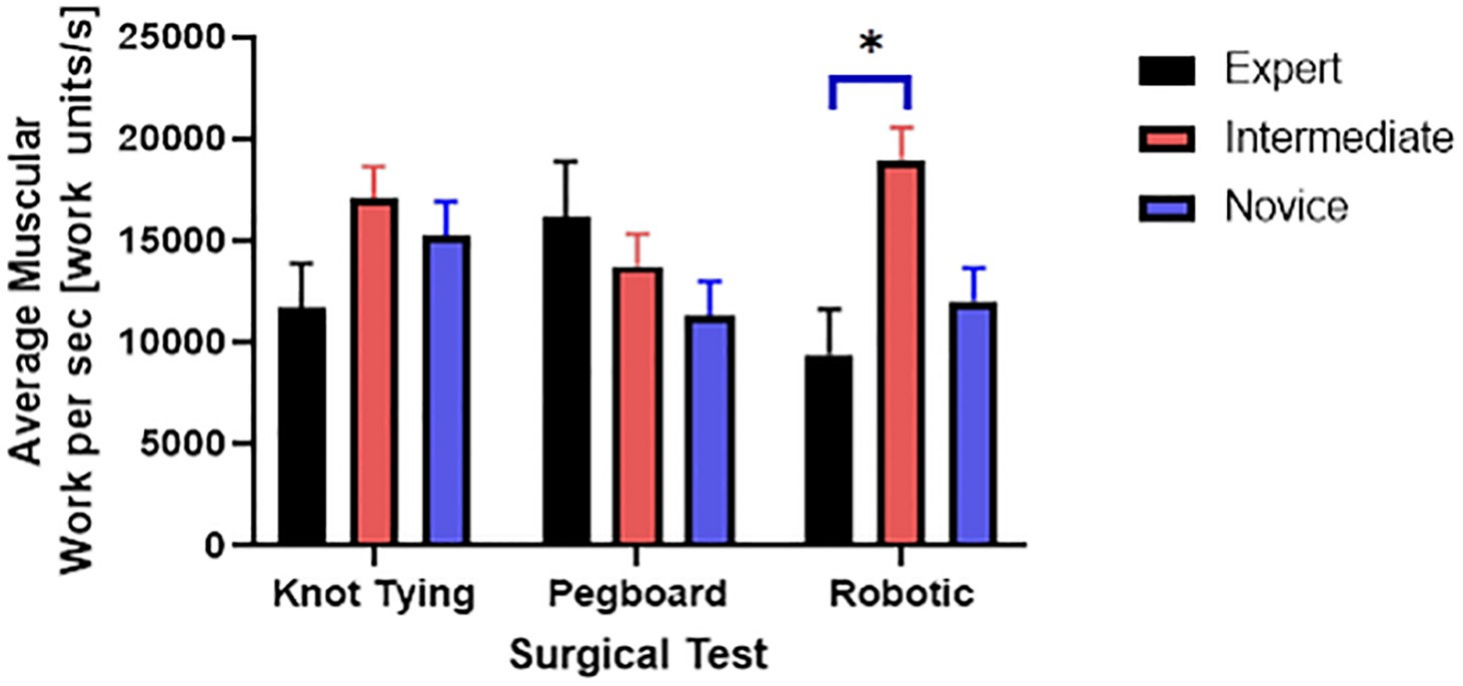
Average muscular work done per second and significant differences in the robotic suturing task.

Post hoc comparisons using the Tukey HSD test indicated that the AWS was significantly lower for the expert group than the intermediate group for the robotic suturing task (M = 9400, SD = 2226 versus M = 18960, SD = 1596) (Fig 9).

**Fig 10:**
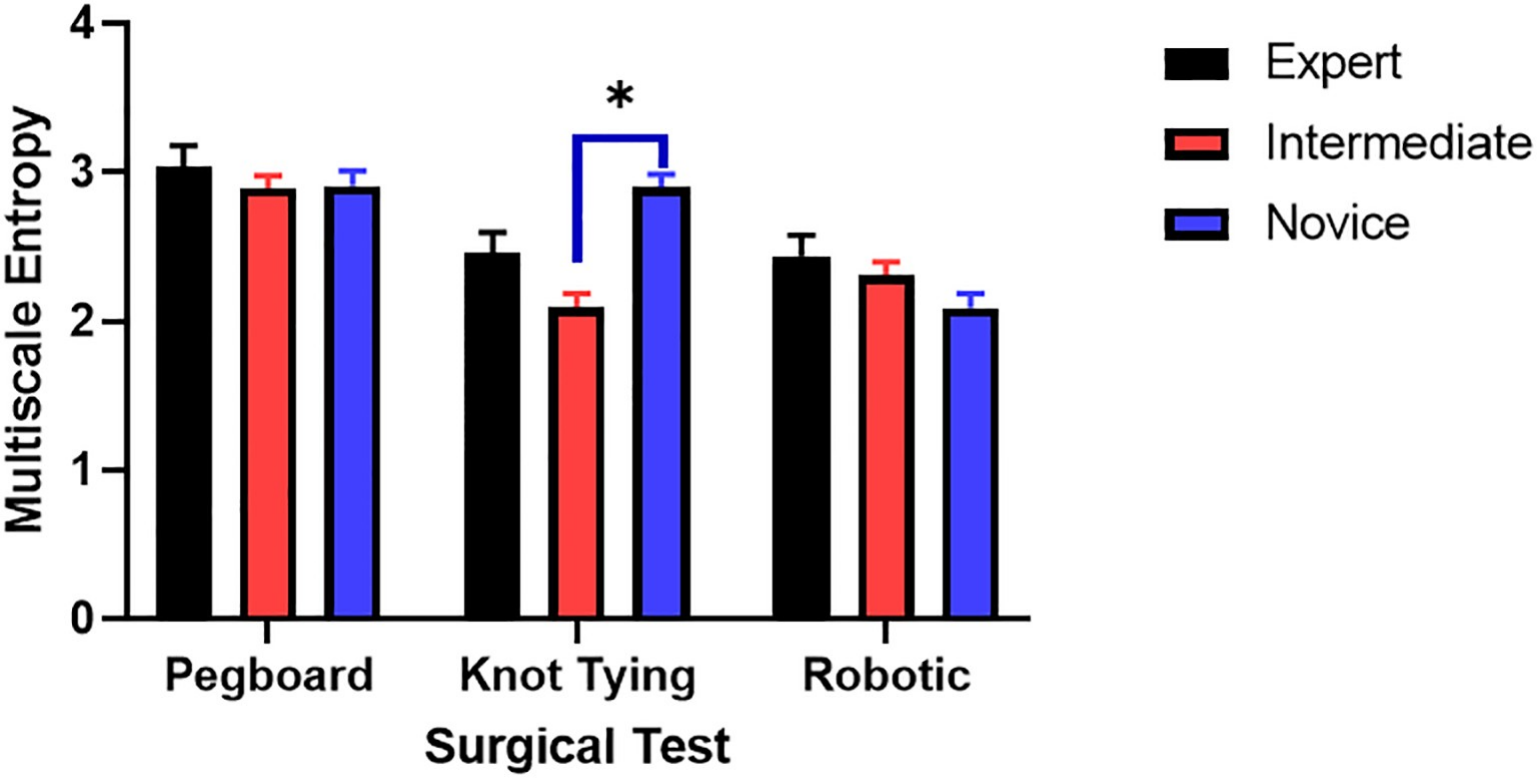
High complexity measured by multiscale entropy.

We also found significant interaction effects among skill level and the three tasks for MSE (F (4,24) = 19.3, p<0.01). Post hoc comparisons using the Tukey HSD test indicated that the MSE was significantly lower for the intermediate (M = 2.09, SD = 0.09) group compared to the novice (M = 2.9, SD = 0.09) group for the knot tying task (Fig 10).

**Fig 11:**
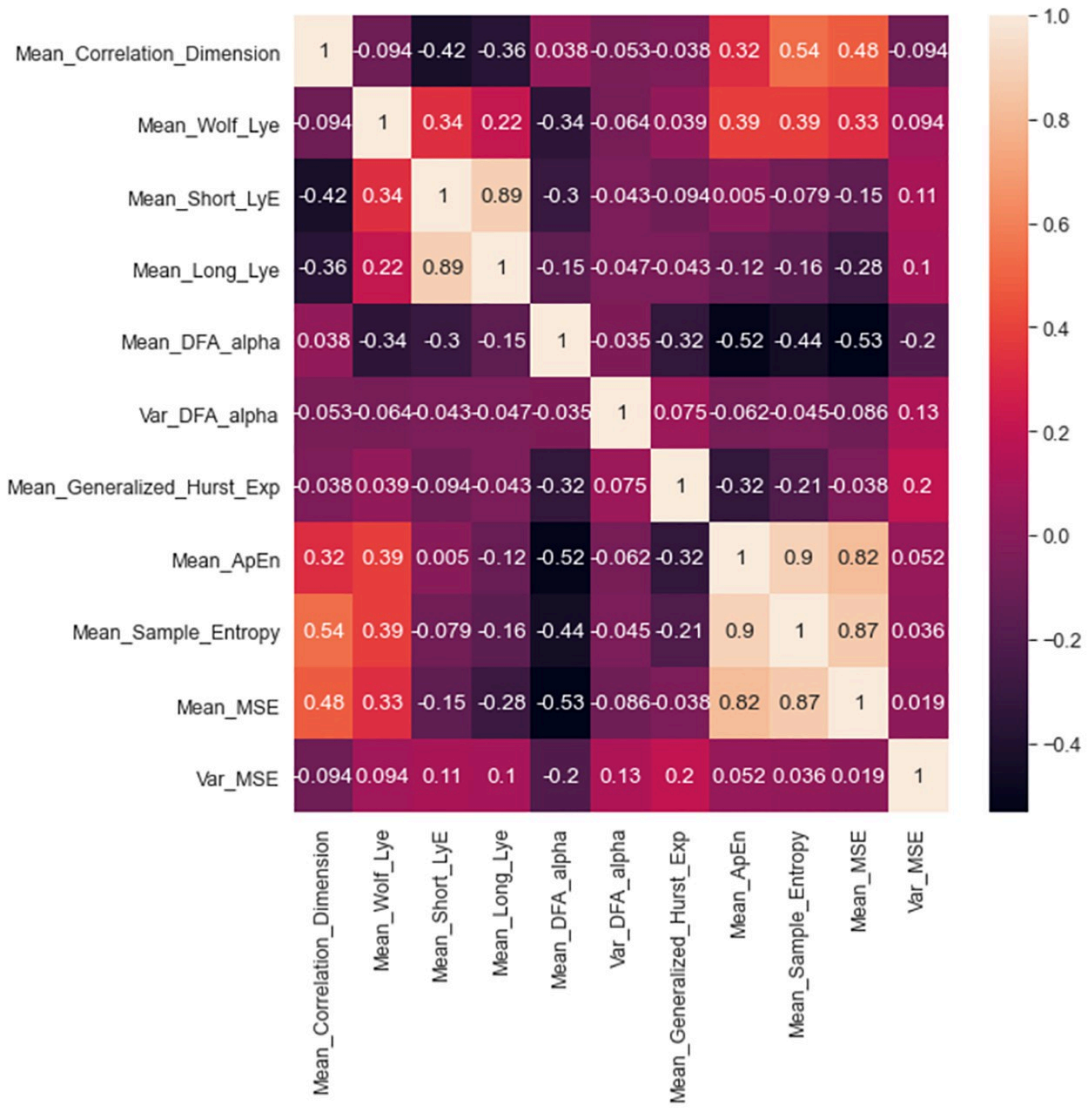
A correlation matrix shows correlation coefficients between features.

Feature selection methods as i) Correlation-based filters (CFS) and ii) recursive feature elimination (RFE) identified important features with high accuracy. Both CFS (Fig 11) and RFE algorithms (Table 3) were tested by implementing machine learning algorithms such as Random Forest classifier, Naïve Bayes, and support vector machines. Inbuilt feature importance methods were utilized for forecasting for the most valuable variables and associated muscles on the model. The models were built using inbuilt feature importance methods, where only significant features are selected, and skill prediction results were evaluated. We found that nonlinear features of complexity such as approximate entropy, multiscale entropy, and sample entropy carried higher importance weightage for accurate classification of surgical skill. We also found that nonlinear features such as complexity as measured by approximate entropy (ApEn), sample entropy (SampEn), and multiscale entropy (MSE) were significantly correlated (Fig 11). We also found long and short Lyapunov exponents were highly correlated.

**Table 3:** Feature importance as determined by recursive feature elimination method.

Feature Priority	Weights
Mean_Apen	0.0453
Mean_MSE	0.0475
Mean_Sample_Entropy	0.0477
Var_MSE	0.0049
Mean_Wolf_Lye	0.0509
Mean_Short_Lye	0.0524
Mean_Long_Lye	0.0544
Mean_Correlation_Dimension	0.0596
Mean_Generalized_Hurst_Exp	0.0635

Prediction accuracies for surgical skill were determined for all six muscles (biceps, triceps, deltoid, ECU, FCU, and TE) for all three machine learning algorithms using selected features (Table 4). Different ML algorithms utilized various muscle-derived features for higher surgical skill classification. We found i) random forest classifier showed higher accuracies with ECU-61%, Deltoid-55%, and TE-55%, ii) support vector machines showed higher accuracies with Deltoid-57%, biceps-45%, ECU-41%, iii) Naïve Bayes showed higher accuracies with biceps-47%, and ECU-43% (Table 4). Since TE location hinders surgical tasks, thus was excluded for further analysis.

**Table 4:** Performance metrics were evaluated by the three ML models (random forest classifier, SVM, and Naïve Bayes) with six muscle datasets. Highest accuracies are heighted in the table as bold.

	Random Forest Classifier				Support Vector Machine				Naive Bayes			
Muscles	Accuracy	Precision	Recall	F1 score	Accuracy	Precision	Recall	F1 score	Accuracy	Precision	Recall	F1 score
Biceps	0.40	0.27	0.33	0.29	0.45	0.46	0.39	0.37	**0.47**	0.46	0.45	0.45
Deltoid	0.55	0.40	0.43	0.41	**0.57**	0.39	0.45	0.41	0.28	0.3	0.25	0.26
ECU	**0.61**	0.73	0.53	0.51	0.41	0.30	0.35	0.30	0.43	0.40	0.41	0.39
FCU	0.45	0.31	0.37	0.32	0.35	0.25	0.30	0.24	0.35	0.25	0.26	0.25
TE	0.55	0.39	0.44	0.40	0.36	0.23	0.29	0.25	0.36	0.32	0.33	0.30
Triceps	0.51	0.36	0.41	0.37	0.53	0.35	0.42	0.38	0.31	0.31	0.28	0.28

Since the three important muscles with the highest accuracies were found to be i) ECU, ii) deltoid and iii) biceps. Our investigation of muscle combinations revealed that the combination of three muscles for skill evaluation resulted in high accuracies for RFC (52%), SVM (50%), and naïve Bayes (40%) algorithm (Table 5). However, combining two muscle locations for sensors such as ECU and deltoid resulted in higher classification accuracy for RFC (58%) and SVM (50%). A combination of ECU and biceps resulted in higher accuracy for the naïve Bayes algorithm (41%) (Table 5).

**Table 5:** This table shows the performance of a combination of muscles for surgical skill evaluation for each model separately. Accuracy, precision, recall, and F1 score are reported for all 3 classification models. Highest accuracies are highlighted in the table as bold.

	Deltoid, ECU, and Biceps				ECU and deltoid				ECU and biceps			
Algorithm	Accuracy	Precision	Recall	F1 score	Accuracy	Precision	Recall	F1 score	Accuracy	Precision	Recall	F1 score
Random Forest	**0.52**	0.54	0.46	0.45	**0.58**	0.46	0.51	0.51	**0.53**	0.47	0.45	0.28
SVM	0.50	0.66	0.43	0.41	0.5	0.33	0.4	0.36	0.45	0.47	0.39	0.37
Naive Bayes	0.41	0.43	0.43	0.43	0.29	0.33	0.33	0.29	0.44	0.43	0.39	0.39

**Fig 12:**
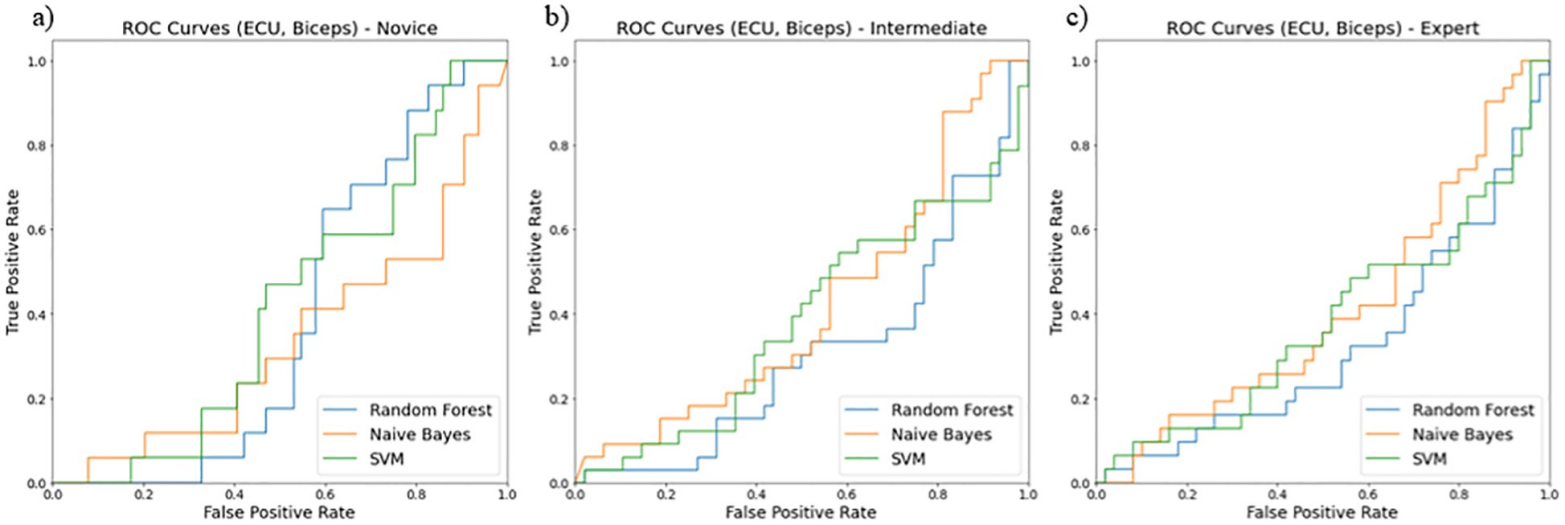
ROC curves showing performance of two muscles (ECU and Biceps) for skill level classification among a) novice, b) intermediate, and c) expert using three classifiers random forest (blue line), Naïve Bayes (orange line), SVM (green line).

**Fig 13:**
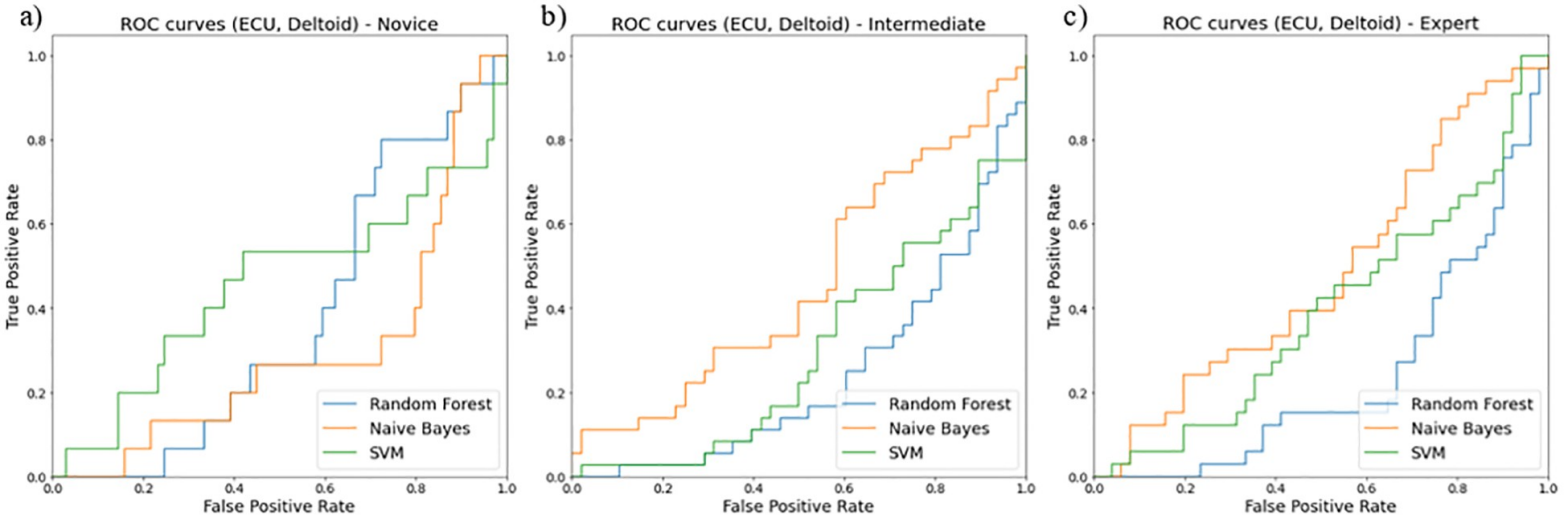
ROC curves showing performance of two muscles (ECU and Deltoid) for skill level classification among a) novice, b) intermediate, and c) expert using three classifiers random forest (blue line), Naïve Bayes (orange line), SVM (green line).

**Fig 14:**
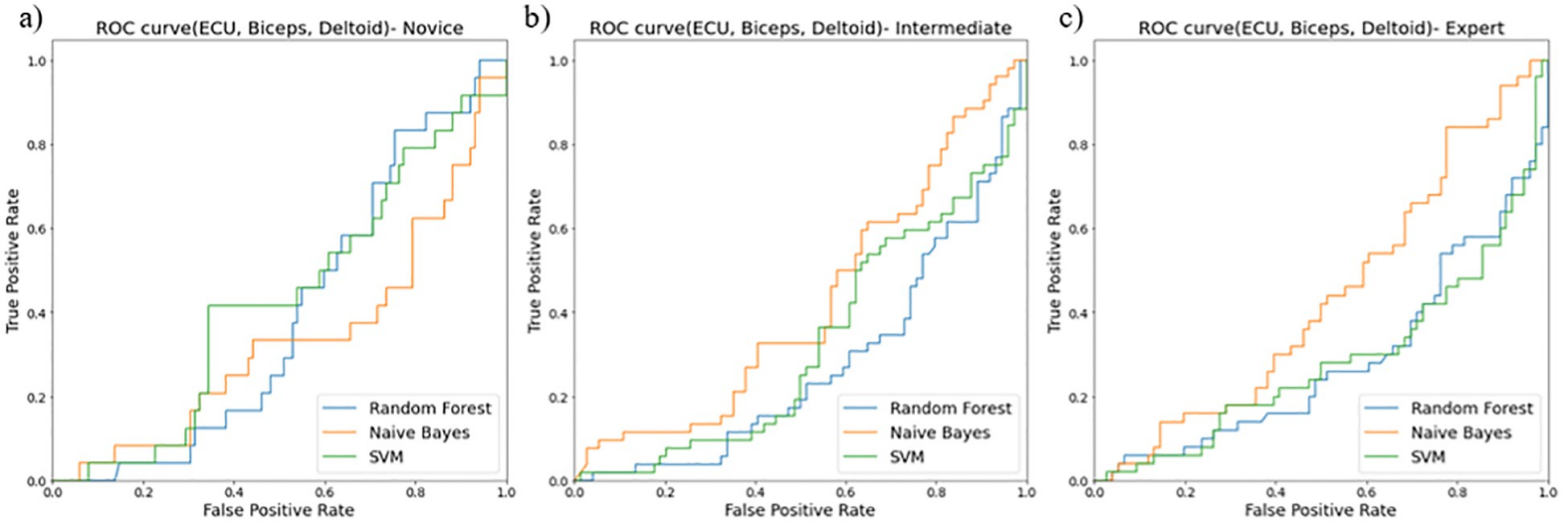
ROC curves showing performance of two muscles (ECU, Biceps and Deltoid) for skill level classification among a) novice, b) intermediate, and c) expert using three classifiers random forest (blue line), Naïve Bayes (orange line), SVM (green line).

The receiver operating curves (ROC) evaluated models for their false positive and true positive rates for three surgical skill classifications (novice, intermediate, and expert). We found the combination of i) ECU and biceps (Fig 12), ii) ECU and deltoid (Fig 13), and iii) ECU, deltoid, and biceps (Fig 14) resulted in the best classification accuracy with minimum sensors on upper extremities. Random forest resulted in the highest accuracy for skill classification among surgeons. We also found muscle ECU and deltoid could achieve an accuracy of 58% during classification.

## Discussion

This work presents machine learning-based methods for selecting the best pairs of sensor features and sensor locations for accurate skill classification during three different surgical tasks. The primary goal of this study was to determine if i) EMG-derived variables such as total time, CMW, AWS, RMS, Range, Frequency, and ii) motion sensor-derived nonlinear variables such as Lyapunov exponent, MSE, correlation dimension can successfully distinguish surgical skill levels. We utilized wearable sensors integrated with EMG and inertial sensors such as accelerometers to classify skill levels in 26 surgeons of three different skill levels (novice, intermediate, and expert). Our work suggests that EMG and accelerometer sensors and nonlinear variability measures can capture the predictability and irregularity of movement fluctuations and thus facilitate surgical skill assessment. Similar studies have adopted data fusion algorithms from wearable inertial and surface EMG sensors to evaluate upper extremity motor function [42].

Our secondary goal was to select the best features and sensor locations for higher classification accuracy. The quality of these selections was tested against features from both right and left limbs and both 3-directional accelerometer and EMG sensors. Nonlinear movement variability features extracted from accelerometer data such as approximate entropy, sample entropy, and multiscale entropy represent a high amount of information to classify skill groups (novice, intermediate, and expert). We also found the most optimal location for sensors to be i) ECU and deltoid, ii) ECU, deltoid, and biceps, and iii) ECU and biceps. These positions resulted in higher accuracy of skill classification among surgeons. As shown in Table 5, for the deltoid, ECU, biceps dataset, the accuracy for RFC is 52% which is 2% more than SVM and 11% more than the Naïve Bayes model. For ECU and deltoid datasets, the accuracy for RFC is about 58% which is 8% more than SVM and 29% more than the Naïve Bayes model. Similarly, for ECU and biceps dataset, the accuracy for RFC is about 53% which is 8% more than SVM and 9% more than the Naïve Bayes model. We discarded the sensor at the TE position since it produces hindrance during surgical tasks and may not be feasible for skill evaluation during surgeries. Our study highlights the importance of nonlinear accelerometer variability measures for skill classification since all accelerometer features showed higher priority using RFE (Table 3).

Training highly skilled and competent surgeons are vital to ensure good quality of patient care and minimize treatment disparities. Surgeons are required to master specific skills during residency and surgical training. However, there is a lack of objective tools utilizing wearable sensors and signal processing which can help differentiate the surgical level of expertise among surgeons. Quantifying and documenting clinical competence and identifying peculiar muscle and movement-based features associated with skill level is a challenging area of research. The studies on clinical competence are limited due to the lack of wearable validated tools. Learning surgical skills involves continuous skills and improvement through feedback from supervising surgeons. Some medical schools have adopted tools like Objective structured assessment of technical skills(OSATS) [5], which are graded by specific criteria like respect for tissue, time and motion, instrument handling, flow in operation, and overall performance [43]. Some other research groups have previously assessed surgical skills utilizing techniques to analyze movement from videos [44–46] and wearable sensors [47–49].

### Statistical differences in features among three skill groups

**Fig 15:**
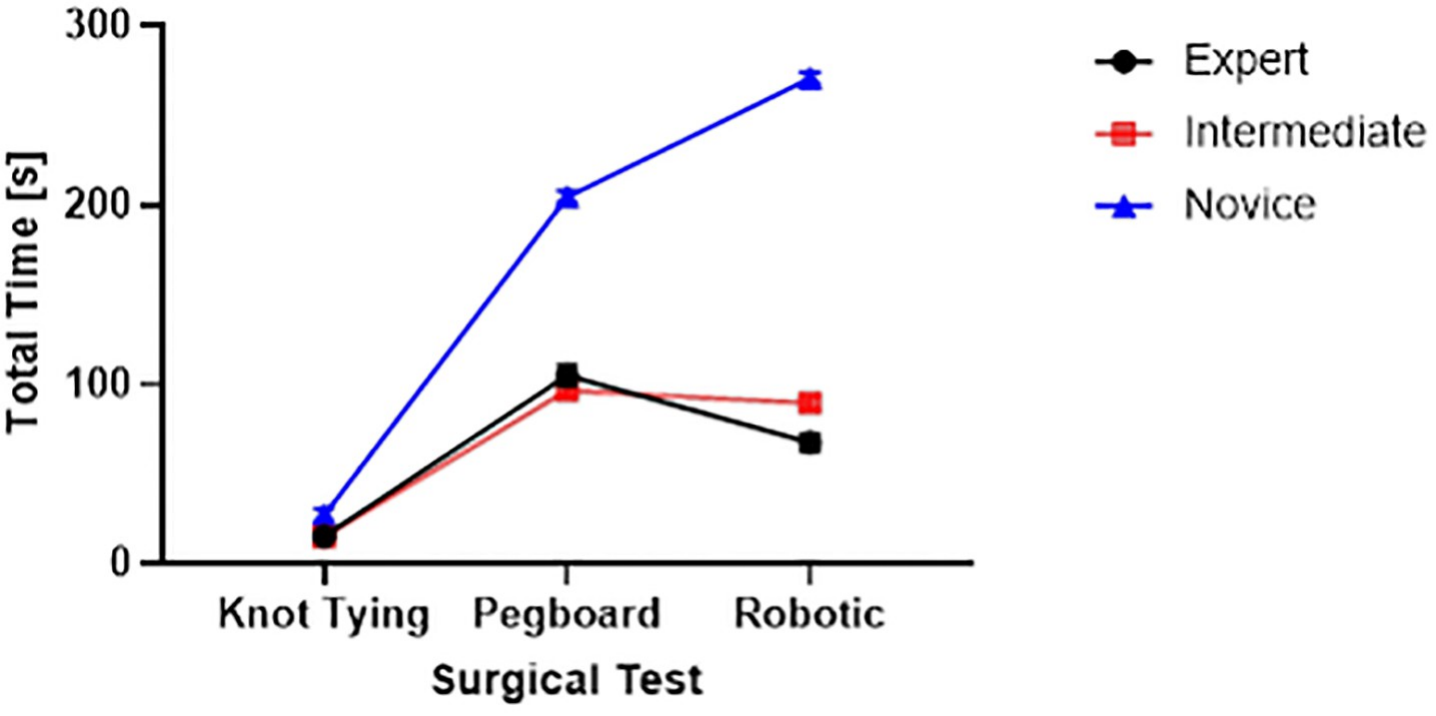
Total time taken for completing surgical tasks (knot tying, pegboard transfer and robotic suturing) by three groups of different skill level (expert, intermediate and novice).

We found that the novice surgeon group took more time to complete the pegboard and robotic suturing task, but not the knot-tying task (Fig 15). Post hoc analysis revealed that the novice group took significantly more time than the expert and intermediate groups for pegboard and robotic suturing tasks. These results suggest that knot tying is equally tricky or straightforward for all groups, thus completing in somewhat similar total times. On the other hand, linear variability measures such as RMS revealed that the expert group had higher muscle firing fluctuations during pegboard transfers, indicating higher adaptability (Fig 6) and task experience. Experts are well trained and may have learned and practiced more than one strategy to transfer cubes during pegboard transfer tasks. Such variability in muscle activations suggests a high degree of freedom and an extensive range of muscle activations among the expert group. We also found muscle firing frequency among surgeons decreased with fewer years of surgical experience (Fig 7). We observed that the novice group had significantly lower muscle firing frequency than the intermediate and expert groups for robotic suturing and knot tying tasks. This could be potentially due to less practice or experience of novice group. Previously researchers have reported that a lower firing rate is observed with impaired muscle function or reduced neuromuscular recruitment [50]. Thus, muscle firing frequency was found to be higher with a high level of surgical expertise. Muscular workload as measured by CMW was significantly higher for the novice group compared to the intermediate group in pegboard transfer. Thus, delineating more muscular work done by novice group compared to intermediate skill group for pegboard transfer task. Besides, CMW was also significantly higher than the expert group in the robotic suturing task (Fig 8). There may be a change in activation patterns after surgical training, with proximal becoming more relaxed and distal muscle groups becoming more active [51], thereby reducing muscular workload or musculoskeletal strain in a surgical expert group compared to novice and intermediate skill groups. When comparing the average rate of muscular work done (work units/s), we found that the intermediate group performed robotic suturing significantly faster than the expert group (Fig 9). This high muscular work rate in the intermediate group may lead to muscle fatigue, contributing to failed surgical procedures.

Correlation-based filter feature selection suggested that nonlinear features such as complexity were significantly correlated (Fig 11) and attained high importance when tested with the recursive feature elimination method (Table 3). High variability in MSE in experts demonstrates that experts can control motor strategies according to task requirements. Higher variability in the correlation dimension of time series is associated with more exchange of dynamical system information among expert groups (Fig 10). Interestingly, previous studies have reported that entropy-based features are more accurate for robotic suturing but not for knot tying [43]. However, the entropy analysis was limited to a single scale using approximate entropy [52]. In this study, we utilized MSE, which provides insights into the complexity of fluctuations over a range of time scales. MSE is advantageous since the time scale of relevance in the surgical task was unknown. MSE could differentiate knot tying skill levels among novice and intermediate groups (Fig 10).

Using features from CFS and RFE allowed identifying important sensor locations that could classify skill levels with higher accuracy. However, we discarded sensor data from the thenar eminence (TE) muscle since it is impractical to conduct surgeries with the sensor at this location due to hindrance. However, ECU, biceps, and deltoid showed the highest accuracy in classification compared to other sites (Tables 4 and 5). These sensor locations are practically feasible and expected to cause the slightest hindrance during surgical tasks-related movements. Our future studies with sensors at these locations will be helpful to build machine learning classification models for surgical skill assessment. An essential contribution of this study is to present new quantitative predictors from muscle activity and movement accelerations to different skill levels in surgeons. This is important over the last decade since increasing sub-specializations and expanded use of MIS techniques have impacted residency training and raised concerns about objectively evaluating residents’ surgical experience or skill level. One of the significant limitations of this study is that it has primarily focused on the surgical task, ignoring mental workload and the support from assistants. Surgical assistants help surgeons, and their interaction is an additional passive task ignored in this study. Future studies are warranted to assess clinical skills better and address these deficits.

Surgical tasks are related to high stress, muscular workload, and variability in surgical task performance. Although objective assessment tools for surgical competence such as EMG and accelerometers are available, information is lacking on important sensor-based features and attachment sites of these wearable systems, which could differentiate surgical skills with the highest accuracy. This study recruited 26 surgeons with three different skill levels and utilized wearable sensors, nonlinear movement variability features, feature selection methods, and classifiers to identify sensor-sites that could distinguish skill levels with the highest accuracy. Advancements in signal processing for movement variability and muscle activation parameters can help develop further assessments. This research can potentially lead to wearable sensor-assisted high fidelity simulation technology, which can evaluate technical training issues with skill decay addressing specific training deficiencies in surgeons. These wearable sensor-based simulation training can assess progress in skill learning or deficits in confidence.

## Supporting information

S1 Table Default ML hyperparameters used for classification. (DOCX)
