# Supplementary material for: Evaluation of surgical skill using machine learning with optimal wearable sensor locations
Source: PLoS One. 2022 Jun 3;17(6):e0267936. doi: 10.1371/journal.pone.0267936 (PMC9165861; doi:10.1371/journal.pone.0267936)
Supplement: S1 Table — (DOCX) [file pone.0267936.s001.docx]

**S1 Table.** Default ML hyperparameters used for classification.

| **RFC** | **Naïve Bayes** | **SVM** |
| --- | --- | --- |
| Bootstrap=True | Variable smoothing = 1e-09 | C=1 |
| Criterion= Gini |  | Cache size=200 |
| Minimum Sample Split=2 |  | Decision Function Shape= one versus rest |
| Minimum sample leaf=1 |  | Degree=3 |
| # of estimators=100 |  | Gamma=scale |
| Warm start=false |  | Kernel=Radial Basis Function |
| Verbose=0 |  | Max iteration=-1 |
|  |  | Shrinking =True |
|  |  | Tolerance=0.001 |
|  |  | Verbose=False |
